# Imipenem-Induced Transcriptional Responses of Porin, Efflux Pumps, and Carbapenemase Genes in Clinical Carbapenem-Resistant *Acinetobacter baumannii*

**DOI:** 10.3390/antibiotics15030299

**Published:** 2026-03-15

**Authors:** Suna Sibel Rizvanoglu, Basar Karaca, Mujde Eryilmaz

**Affiliations:** 1 Department of Pharmaceutical Microbiology, Faculty of Pharmacy, Ankara University, Ankara 06560, Türkiye; sbl.gurpinar@gmail.com; 2 Department of Biology, Faculty of Science, Ankara University, Ankara 06100, Türkiye; karaca@ankara.edu.tr; 3 Department of Pharmaceutical Microbiology, Faculty of Pharmacy, Acibadem Mehmet Ali Aydinlar University, Istanbul 34752, Türkiye

**Keywords:** *Acinetobacter baumannii*, carbapenem resistance, efflux pump, gene expression, imipenem, outer membrane porins

## Abstract

**Background/Objectives**: Carbapenem-resistant *Acinetobacter baumannii* poses a critical threat due to its ability to acquire multiple resistance mechanisms and persist under antibiotic pressure. This study aimed to elucidate the molecular basis of imipenem resistance in clinical *A. baumannii* isolates by integrating phenotypic, molecular, transcriptional, and clonal analyses. **Methods**: Eleven *A. baumannii* isolates identified by MALDI-TOF MS (matrix-assisted laser desorption ionization time-of-flight mass spectrometry) were investigated. Antimicrobial susceptibility to imipenem and meropenem was assessed, followed by polymerase chain reaction (PCR) detection of Ade efflux pump, outer membrane porin, and OXA-type carbapenemase genes. Transcriptional responses to sub-inhibitory imipenem exposure were evaluated using quantitative real-time PCR, and clonal relatedness was assessed by arbitrarily primed PCR. **Results**: All isolates were carbapenem-resistant, with *bla_OXA-23_* detected in all isolates and *bla_OXA-24_* absent in one isolate. Transcriptional analysis revealed isolate-specific responses to imipenem exposure. Among Ade efflux pump components, only *adeR* exhibited expression changes, displaying either downregulation or upregulation depending on the isolate, whereas *adeA*, *adeB*, *adeC*, and *adeS* transcripts were not detected under the tested conditions. Outer membrane porin genes showed heterogeneous regulation, with *ompA* and *carO* downregulated, while some isolates showed increased expression. Expression of *oprD* varied among isolates, and *omp33–36* transcripts were detected in a single isolate and were reduced after exposure. Clonal analysis identified nine distinct genotypes, indicating genetic diversity and the absence of clonal dominance. **Conclusions**: These findings highlight the multifactorial and heterogeneous nature of carbapenem resistance in *A. baumannii*, emphasizing the interplay between regulatory efflux mechanisms, porin modulation, and carbapenemase carriage.

## 1. Introduction

Antimicrobial resistance is currently recognized as one of the most critical global public health challenges, threatening the prevention and treatment of an increasing range of infections. The World Health Organization (WHO) reports that global surveillance data indicate a concerning and sustained increase in resistance rates among major bacterial pathogens, severely compromising the efficacy of last-resort antibiotics. The WHO Global Antibiotic Resistance Surveillance Report highlights that resistance to commonly used antibiotics continues to increase worldwide, with particularly high resistance levels reported for carbapenems, fluoroquinolones, and third-generation cephalosporins [1].

*Acinetobacter baumannii* has become a major cause of hospital-acquired infections worldwide and is listed as a critical priority pathogen on the WHO 2024 Bacterial Priority Pathogens List [2]. *A. baumannii* is associated with severe infections such as pneumonia, bacteremia, meningitis, and wound and urinary tract infections, particularly in critically ill or immunocompromised patients. Its remarkable ability to survive harsh environmental conditions, including desiccation and extreme pH, allows it to persist on hospital surfaces and medical equipment, contributing to its successful nosocomial transmission and complicating infection control [3,4].

Since the 1970s, *A. baumannii* has been recognized as a significant clinical threat due to its remarkable ability to rapidly acquire resistance to a broad spectrum of antibiotics, including carbapenems, which are often considered last-line agents for multidrug-resistant infections [4]. The adaptability of Acinetobacter species to adverse environmental pressures has accelerated the emergence of multidrug-resistant strains worldwide [5]. Carbapenems, a class of β-lactam antibiotics, are generally highly effective against bacteria producing β-lactamases and extended-spectrum β-lactamases and are frequently used as first-line therapy for severe infections. However, the increasing prevalence of carbapenem-resistant *A. baumannii* has become a major clinical and economic burden on healthcare systems, severely limiting therapeutic options [4,6]. In response to this growing threat, the European Centre for Disease Prevention and Control (ECDC) has launched a comprehensive genomic surveillance program across Europe to monitor and track the spread of carbapenem-resistant *A. baumannii* [7].

Resistance to carbapenems in *A. baumannii* is multifactorial, involving both intrinsic and acquired mechanisms. These include enzymatic degradation of antibiotics, modification or protection of target sites, reduced outer membrane permeability, and active efflux of antimicrobial agents [8]. Genotypic resistance may result from horizontal gene transfer or mutations affecting gene regulation, expression, or protein structure. Among these mechanisms, alterations in outer membrane proteins, overexpression of efflux pump systems, and production of OXA-type carbapenemases play central roles in carbapenem resistance [4,6].

Outer membrane porins such as OmpA, OmpW, Omp33–36, CarO, and OprD are critical determinants of antibiotic permeability in *A. baumannii*. OmpA contributes to outer membrane stability and biofilm formation, enhancing bacterial survival and antibiotic tolerance [9]. Reduced expression or structural alterations of Omp33–36 and CarO are associated with decreased carbapenem influx and reduced susceptibility, particularly to imipenem [10,11,12]. CarO inactivation or loss is frequently reported in carbapenem-resistant strains and results in impaired drug entry [11]. In addition, OprD homologues in *A. baumannii*, similar to those in *P. aeruginosa*, are implicated in carbapenem transport and resistance modulation [3].

Efflux pump systems further contribute to the multidrug-resistant phenotype of *A. baumannii*. The AdeABC resistance-nodulation-cell division efflux system, encoded by *adeA*, *adeB*, and *adeC* and regulated by *adeR* and *adeS*, actively exports a wide range of antibiotics, reducing intracellular drug concentrations [8,12,13]. Mutations in the regulatory genes *adeR* and *adeS* can result in overexpression of the efflux system and are frequently associated with multidrug resistance [11]. Additionally, the production of OXA-type β-lactamases, particularly OXA-23 and OXA-24, remains one of the most important mechanisms driving carbapenem resistance by hydrolysing carbapenem antibiotics [14].

Despite extensive documentation of resistance gene carriage in *A. baumannii*, the isolate-specific transcriptional adaptation of porin- and efflux-associated genes after carbapenem exposure remains insufficiently understood, particularly under sub-inhibitory antibiotic pressure. Understanding these expression dynamics is critical for elucidating the adaptive mechanisms that contribute to carbapenem resistance beyond gene acquisition. Therefore, this study aimed to investigate imipenem resistance in clinical *A. baumannii* isolates by analyzing the expression levels of selected outer membrane porin genes, efflux pump regulatory genes, and OXA-type carbapenemase genes following imipenem exposure, as well as evaluating the clonal relatedness of the isolates.

## 2. Results

### 2.1. Antibiotic Susceptibility of the Clinical Isolates

Carbapenem susceptibility of *A. baumannii* isolates was confirmed by the disk diffusion test. All isolates were resistant to imipenem and meropenem (Table S1).

Before imipenem exposure, the minimum inhibitory concentration (MIC) values of imipenem for the *A. baumannii* isolates were determined. Interpretation criteria for imipenem susceptibility were defined as ≤2 µg/mL for susceptible isolates and ≥4 µg/mL for resistant isolates. The MIC values of the tested isolates are presented in Table 1. According to the MIC results, all isolates were resistant to imipenem. *A. baumannii* ATCC 1709 and *A. baumannii* ATCC 1799 were included as reference strains in the assays. These MIC values were used to define sub-inhibitory imipenem concentrations (½ MIC) for subsequent gene expression analysis.

### 2.2. Detection of Efflux Pump, Outer Membrane Porin, and Carbapenemase Genes by PCR

The presence of Ade efflux pump genes (*adeA*, *adeB*, *adeC*, *adeR*, and *adeS*), outer membrane porin genes (*ompA*, *ompW*, *omp33–36*, *carO*, and *oprD*), and carbapenemase resistance genes (*bla_OXA-23_* and *bla_OXA-24_*) was investigated by PCR in the *A. baumannii* isolates.

Agarose gel electrophoresis revealed clear, specific amplification products at the expected sizes for all target genes. The *adeA*, *adeB*, *adeC*, *adeR*, and *adeS* genes yielded PCR products of 513 bp, 981 bp, 560 bp, 314 bp, and 660 bp, respectively, confirming the presence of the AdeABC efflux system and its regulatory components. Amplification of outer membrane porin genes produced products of 86 bp (*ompA*), 543 bp (*ompW*), 96 bp (*carO*), 194 bp (*omp33–36*), and 134 bp (*oprD*), indicating the widespread presence of porin-associated genes among the isolates. For carbapenemase genes, PCR amplification of *bla_OXA-23_* produced a distinct 128 bp band in all tested isolates, confirming the presence of the *bla_OXA-23_* carbapenemase gene. In contrast, the *bla_OXA-24_* gene (142 bp) was detected in all isolates except isolate 1, indicating its absence in this isolate and presence in the others. These PCR results demonstrate the concurrent presence of multiple efflux pump genes, outer membrane porins, and carbapenem resistance determinants in the analyzed isolates, highlighting the multifactorial genetic basis of antimicrobial resistance (Figure S1).

### 2.3. Quantitative Determination of Antibiotic Resistance Genes mRNA Expression

The expression changes in outer membrane porin genes (*ompA*, *ompW*, *omp33–36*, *carO*, and *oprD*), Ade efflux pump genes (*adeA*, *adeB*, *adeC*, *adeR*, and *adeS*), and OXA-type carbapenemase genes (*bla_OXA-23_* and *bla_OXA-24_*) were evaluated using mRNA obtained from selected *A. baumannii* isolates before and after imipenem exposure. Comparative analysis revealed differential regulation of porin and efflux pump-associated genes after antibiotic exposure. The quantified relative expression levels of all analyzed genes are shown in Table 2.

Among the analyzed isolates, *adeR* was the only gene of the Ade efflux pump system that showed a measurable change in expression following imipenem exposure. The *adeR* gene was downregulated in isolates 3, 4, 5, and 6, while upregulation was detected in isolates 2, 7, 10, and 11 (Table 2). In contrast, expression of the other Ade efflux pump genes (*adeA*, *adeB*, *adeC*, and *adeS*) was not detected in any of the isolates. Similarly, *ompW*, *bla_OXA-23_*, and *bla_OXA-24_* expression was not detected under the tested conditions. For outer membrane porin genes, *ompA* and *carO* expression levels generally decreased after imipenem exposure; however, increased expression of both genes was observed in isolates 10 and 11 (Table 2). Expression of *oprD* showed isolate-dependent variation, with notable upregulation in isolate 10 following exposure. *omp33–36* expression remained largely unchanged across most isolates; however, a marked downregulation was observed in isolate 11 following imipenem exposure. Overall, quantitative real-time PCR analysis demonstrated distinct, isolate-specific transcriptional profiles in response to imipenem exposure.

**Figure 1:**
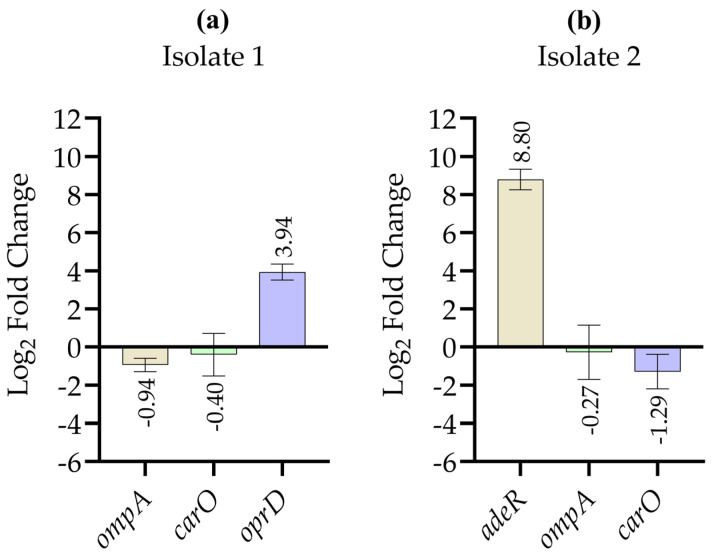
Log_2_-transformed relative expression levels of selected resistance-associated genes in *A. baumannii* isolates after imipenem exposure, determined by quantitative real-time PCR. (**a**) Log2 fold change of *ompA*, *carO*, and *oprD* in isolate 1; (**b**) log_2_ fold change of *adeR*, *ompA*, and *carO* in isolate 2. Relative expression values were calculated using the 2^−ΔΔCt^ method after normalization to the 16S rRNA housekeeping gene and then log_2_ transformed for graphical representation. Data are presented as mean ± standard deviation. RT-qPCR measurements were performed in technical duplicates for each condition, and results are shown as relative expression trends.

Figure 1a shows the relative expression levels of *ompA*, *carO*, and *oprD* in isolate 1 of *A. baumannii*, comparing imipenem-treated samples with the corresponding non-treated control group. Expression levels of *ompA* and *carO* showed no clear differences between treated and non-treated conditions. In contrast, a marked increase in *oprD* expression was observed after imipenem exposure compared with the non-treated control. Figure 1b shows the relative expression levels of *adeR*, *ompA*, and *carO* in isolate 2 of *A. baumannii*, with values normalized to the non-treated control group. A pronounced upregulation of *adeR* expression was detected after imipenem exposure, while no clear differences in *ompA* or *carO expression* were observed between treated and non-treated samples.

**Figure 2:**
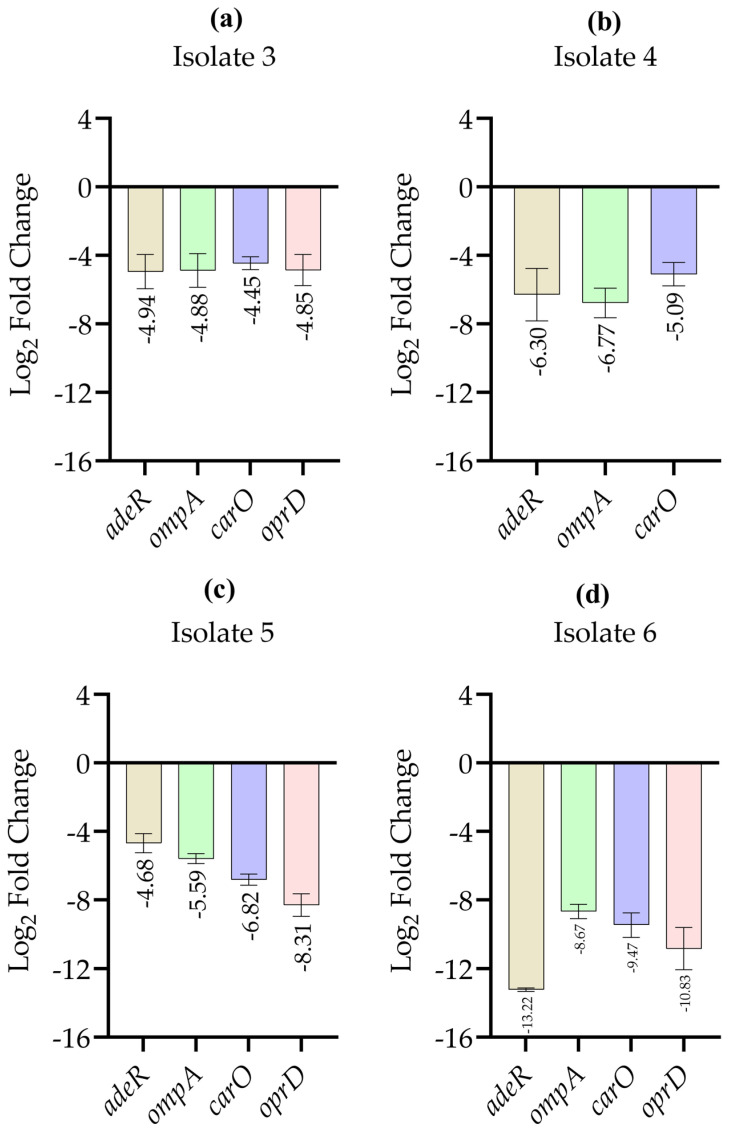
Log_2_-transformed relative expression levels of selected resistance- and permeability-associated genes in *A. baumannii* isolates following imipenem exposure, determined by quantitative real-time PCR. Panels (**a**–**d**) represent isolates 3, 4, 5, and 6, respectively. Log_2_ fold changes of *adeR*, *ompA*, *carO*, and *oprD* are shown relative to the corresponding untreated control groups. *oprD* expression was not detectable in isolate 4 under the tested experimental conditions. Relative expression values were calculated using the 2^−ΔΔCt^ method after normalization to the 16S rRNA housekeeping gene and then log_2_ transformed for graphical representation. Data are presented as mean ± standard deviation. RT-qPCR measurements were performed in technical duplicates for each condition, and results are shown descriptively as relative expression trends.

Figure 2 shows the relative expression levels of selected resistance and permeability-associated genes in *A. baumannii* isolates 3–6 following imipenem exposure compared with their respective non-treated control groups. In all analyzed isolates, imipenem treatment resulted in marked downregulation of *adeR*, *ompA*, *carO*, and *oprD* relative to controls, demonstrating a similar pattern within this isolate set transcriptional response across these isolates.

**Figure 3:**
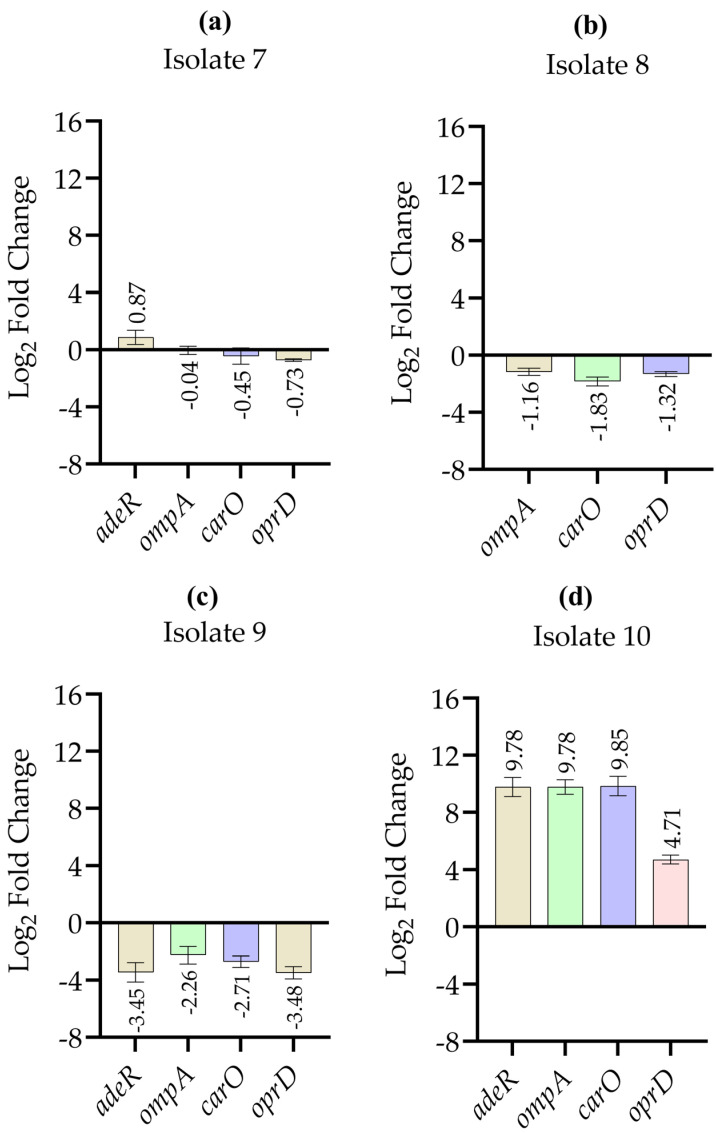
Log_2_-transformed relative expression levels of selected resistance- and permeability-associated genes in *A. baumannii* isolates following imipenem exposure, determined by quantitative real-time PCR. Panels (**a**–**d**) represent isolates 7, 8, 9, and 10, respectively. Log_2_ fold changes of *adeR*, *ompA*, *carO*, and *oprD* are shown relative to the corresponding untreated control groups. *adeR* expression was not detectable in isolate 8 under the tested experimental conditions. Relative expression values were initially calculated using the 2^−ΔΔCt^ method after normalization to the 16S rRNA housekeeping gene and then log_2_ transformed for graphical representation. Data are presented as mean ± standard deviation. RT-qPCR measurements were performed in technical duplicates for each condition, and results are presented descriptively as relative expression trends.

Figure 3 depicts the relative expression levels of resistance and permeability-associated genes in *A. baumannii* isolates 7–10 after imipenem exposure compared with untreated control groups. In isolate 7, imipenem exposure altered the expression levels of *adeR*, *ompA*, *carO*, and *oprD*; however, the observed changes were not pronounced. In isolate 8, imipenem exposure resulted in a marked downregulation of *carO* expression, while changes in *ompA* and *oprD* were not pronounced. In isolate 9, imipenem treatment was associated with coordinated downregulation of *adeR*, *ompA*, and *carO* compared to the untreated control. In contrast, isolate 10 showed markedly increased fold-change values for *adeR*, *ompA*, and *carO* after imipenem exposure compared to the untreated control.

**Figure 4:**
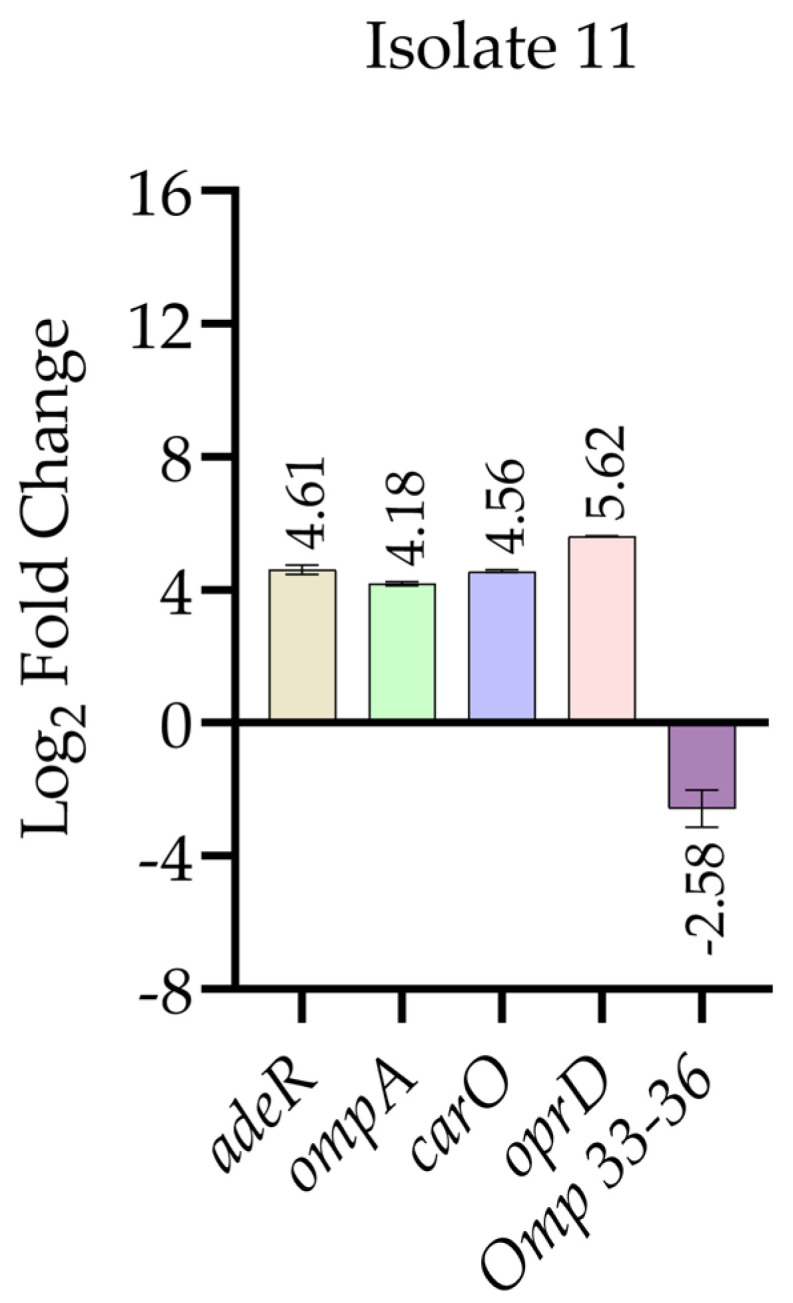
Log_2_-transformed relative expression levels of selected resistance- and permeability-associated genes in *A. baumannii* isolate 11 following imipenem exposure, determined by quantitative real-time PCR. Log_2_ fold changes in *adeR*, *ompA*, *carO*, *oprD*, and *Omp33–36* are shown relative to the corresponding non-treated control group. Relative expression values were initially calculated using the 2^−ΔΔCt^ method after normalization to the 16S rRNA housekeeping gene and subsequently log_2_ transformed for graphical representation. Data are presented as mean ± standard deviation. RT-qPCR measurements were performed in technical duplicates for each condition, and results are presented descriptively as relative expression trends.

Figure 4 presents the relative expression levels of selected resistance- and permeability-associated genes in *A. baumannii* isolate 11 following imipenem exposure compared with the corresponding non-treated control group. Quantitative real-time PCR analysis revealed a pronounced transcriptional response after antibiotic treatment. Expression levels of *adeR*, *ompA*, *carO*, and *oprD* were markedly increased in the imipenem-treated group relative to the non-treated control. In contrast, *omp33–36* expression was markedly reduced following imipenem exposure compared with the control condition.

### 2.4. Arbitrarily Primed PCR (AP-PCR)

The AP-PCR dendrogram illustrates the genetic relatedness among the analyzed *A. baumannii* isolates based on similarities in their amplification banding patterns. Cluster analysis grouped the isolates into nine distinct genotypes, indicating a high level of genetic diversity within the isolate collection. The similarity coefficients ranged from approximately 39.8% to 100%, reflecting the presence of both closely related and genetically distinct strains.

**Figure 5:**
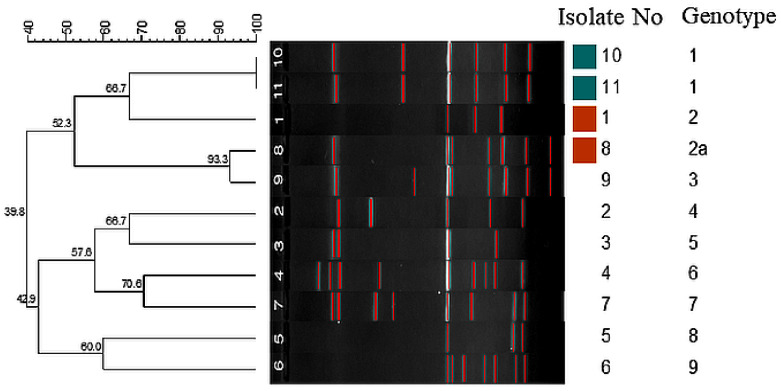
Dendrogram obtained from AP-PCR results for *A. baumannii* isolates.

Isolates 10 and 11 clustered together within the same genotype and exhibited a high degree of genetic similarity, suggesting clonal relatedness. In contrast, isolates 1, 8, and 9 were assigned to separate genotypes, indicating distinct genetic backgrounds. The remaining isolates from 2 to 7 were distributed across individual genotypes, further supporting the presence of multiple unrelated lineages. The absence of a dominant cluster containing the majority of isolates suggests that the analyzed *A. baumannii* population is genetically heterogeneous rather than originating from a single clonal source. Overall, the AP-PCR results reveal substantial genomic variability among the isolates and demonstrate the coexistence of multiple genotypes within the study population (Figure 5).

## 3. Discussion

Carbapenem-resistant *A. baumannii* (CRAB) causes serious hospital-acquired infections and alarmingly high mortality, particularly among intensive care unit patients with prolonged hospitalization. Its persistence in the hospital setting and rapid acquisition or combination of multiple resistance markers cause outbreaks that are difficult to control and have limited treatment options. WHO has included CRAB among the critical-priority bacterial pathogens for research and development on its 2024 Bacterial Priority Pathogens List. It emphasizes that antibiotic resistance in these pathogens severely erodes treatment options and must be combated with new strategies [2]. Furthermore, the European Centre for Disease Prevention and Control (ECDC) has highlighted CRAB as a priority pathogen for genomic-based research and strategy-focused surveillance, due to ongoing concerns about transmission dynamics and the evolution of resistance [7].

Although OXA-type carbapenemases, porin alterations, and efflux pumps are known to contribute to carbapenem resistance in CRAB, the combined transcriptional responses of these mechanisms under antibiotic exposure remain poorly understood. Recent studies have shown that *A. baumannii* employs a multifactorial, adaptive resistance network rather than a single dominant mechanism, allowing effective survival under antibiotic exposure [15,16]. Therefore, addressing multidrug-resistant infections requires a holistic understanding of how distinct resistance pathways interact and respond to antimicrobial stress. Accordingly, our study aimed to investigate adaptive expression changes in these resistance systems in clinical isolates exposed to sub-MIC imipenem concentrations. In the present design, isolates were subjected to prolonged (16 h) sub-MIC exposure before RNA extraction. Therefore, the observed transcriptional profiles likely represent stabilized adaptive states under sustained antibiotic pressure rather than early, transient stress responses. It should be noted that acute transcriptional dynamics occurring within minutes to a few hours after exposure were not captured in this study. We analyzed the expression of outer membrane porin genes (*ompA*, *ompW*, *carO*, *omp33–36*, and *oprD*), efflux pump components (*adeA*, *adeB*, *adeC*, *adeR*, *adeS*), and OXA-type carbapenemase genes (*bla_OXA-23_* and *bla_OXA-24_*) in imipenem-resistant clinical *A. baumannii* isolates before and after imipenem exposure, and evaluated their genotypic diversity by AP-PCR.

The role of the AdeABC efflux system in carbapenem resistance remains controversial. Although efflux pumps alone rarely confer high-level resistance, they can significantly contribute when combined with other resistance mechanisms [8,17]. Recent studies have emphasized the importance of two-component regulatory systems, showing that efflux-associated resistance may be linked to *adeABC* overexpression driven by alterations in regulatory genes and associated mobile genetic elements, such as ISA*ba1*-containing resistance islands [18]. These findings suggest that regulatory and genomic context strongly influence the detectability and contribution of *adeABC* expression across different strain collections and experimental conditions.

Our results showed that only the response regulator gene *adeR* exhibited measurable transcriptional changes in the isolates. In contrast, the structural efflux pump genes (*adeA*, *adeB*, *adeC*) and the sensor kinase gene (*adeS*) were transcriptionally undetectable under the tested conditions. The absence of a detectable transcriptional response from the structural Ade efflux system genes may therefore be associated with mutations or structural alterations in the AdeRS regulatory system and related genomic regions, as suggested by Słoczyńska et al. [18]. However, further molecular and genomic analyses are required to verify these potential mechanisms.

In *A. baumannii*, reduced outer membrane permeability is considered a key adaptation mechanism in the development of antimicrobial resistance, particularly in the emergence of carbapenem resistance or decreased carbapenem susceptibility. Recent reviews emphasize that carbapenem resistance is not limited to carbapenemase production; instead, it develops within a multifactorial resistance mechanism shaped by the interplay of outer membrane proteins, efflux systems, and regulatory systems [15,16]. In this context, decreased expression of porins such as CarO, Omp33–36, and OprD restricts the entry of antibiotics into the cell, reducing intracellular antibiotic concentration and contributing to the emergence of the resistance phenotype [6].

CarO protein is recognized as a key porin that facilitates the transport of carbapenems through the outer membrane into the cell. Recent studies provide compelling evidence that CarO loss or structural alteration contributes to imipenem resistance in *A. baumannii*. Zhu et al. [19] demonstrated that CarO-deficient clinical isolates exhibited up to a 16-fold increase in imipenem MIC, which was fully reversed upon genetic complementation, establishing a direct functional link between CarO loss and carbapenem resistance. In parallel, structural modeling studies by Gopikrishnan et al. [20] revealed that mutations in the imipenem-resistant CarO porin constrict the channel structure and impair carbapenem permeability, further supporting the central role of CarO in mediating carbapenem uptake. These findings highlight the central role of CarO in carbapenem resistance. In our study, a marked decrease in *carO* expression levels was observed in the majority of isolates after imipenem exposure, consistent with the literature.

Omp33–36 and OprD are among the outer membrane porins associated with carbapenem resistance in *A. baumannii*. Multiple studies have consistently reported that decreased expression or loss of *omp33–36* and *oprD*, often together with *carO*, represents a characteristic permeability-related mechanism contributing to carbapenem resistance across diverse clinical isolates and geographical regions [21,22,23].

In addition to the loss of classical porins such as CarO, Omp33–36, and OprD, recent studies suggest that non-classical outer membrane proteins, including OmpA and OmpW, may also influence antibiotic tolerance and resistance phenotypes in *A. baumannii*. OmpA, one of the most abundant outer membrane proteins, has been described as a multifunctional protein involved in virulence, host interaction, and stress tolerance [24]. Consistent with this, Δ*ompA* mutants have been reported to exhibit increased susceptibility to multiple antibiotics, including imipenem [25]. These findings indicate that OmpA may contribute to the resistance phenotype indirectly, by supporting membrane integrity and cellular stress adaptation, rather than acting as a classical permeability-limiting porin.

Although the precise role of *ompW* in carbapenem resistance remains unclear, available evidence suggests that OmpW functions as a selective small-molecule channel involved in membrane homeostasis and metabolic adaptation under stress, rather than as a primary determinant of carbapenem permeability [26,27]. Consistent with the concept that carbapenem resistance arises from a multifactorial adaptive network rather than a single dominant mechanism [16], non-classical outer membrane proteins such as OmpA and OmpW may contribute indirectly to resistance by supporting stress tolerance and membrane stability. In this context, our data revealed heterogeneous regulation of *ompA* following imipenem exposure, whereas *ompW* was detected at the genomic level but showed no measurable transcriptional response. These findings suggest that the contributions of OmpA and OmpW to carbapenem resistance are likely context-dependent and indirect, acting alongside permeability reduction, regulatory adaptation, and other resistance mechanisms.

The acquired carbapenemase gene *bla_OXA-23_* is widely disseminated among carbapenem-resistant *A. baumannii* isolates and remains the dominant OXA-type carbapenemase worldwide, whereas *bla_OXA-24/40_* variants are generally less frequent [22,28]. However, carbapenem resistance cannot be explained solely by the presence of OXA genes, as their contribution to phenotypic resistance largely depends on transcriptional activation. Previous studies have demonstrated that insertion of the IS*Aba1* element upstream of *bla*OXA genes provides strong promoter activity, leading to high-level expression of both intrinsic and acquired OXA-type carbapenemases and constituting a major driver of carbapenem resistance [29,30]. In the present study, *bla_OXA-23_* was detected by PCR in all isolates and *bla_OXA-24_* in all but one isolate; however, transcriptional expression of these genes was not detected under the tested conditions. As the presence of ISA*ba1* upstream of *bla*OXA genes and the broader promoter architecture were not investigated, the extent to which ISA*ba1*-mediated overexpression contributes to the observed resistance phenotype could not be assessed. Only *bla_OXA-23_* and *bla_OXA-24_* were screened; therefore, the potential contribution of other carbapenemase genes or additional OXA variants cannot be ruled out. This represents a limitation of the present study and warrants further investigation that incorporates genomic context analysis.

In light of these findings, the 11 clinical isolates exhibited heterogeneous transcriptional responses to imipenem exposure, suggesting adaptive variability among these isolates. Rather than a uniform response, the isolates displayed distinct transcriptional patterns involving differential regulation of porin-associated genes and the efflux regulator *adeR*, consistent with a potentially multifactorial transcriptional response profile under the tested conditions. A subset of isolates (3, 4, 5, 6, and 9) exhibited simultaneous downregulation of *ompA*, *carO*, and *oprD*, accompanied by reduced *adeR* expression. This transcriptional pattern may reflect a permeability-associated transcriptional adjustment under imipenem exposure. However, given the small sample size, these findings should be interpreted cautiously and require validation in larger and genetically diverse isolate collections. In isolate 8, *adeR* transcripts were undetectable both before and after imipenem exposure, whereas *carO* expression was markedly downregulated. This profile indicates that modulation of membrane permeability alone may contribute to the adaptive response under imipenem stress. Isolate 1 displayed a distinct response characterized by marked upregulation of *oprD* following exposure. This pattern suggests isolate-specific modulation of porin-associated transcription; however, the functional consequences of this response remain to be determined. In contrast, isolate 2 exhibited pronounced upregulation of *adeR* after imipenem exposure, potentially reflecting a regulatory adaptive response rather than a primary permeability reduction strategy. Finally, isolates 10 and 11 demonstrated coordinated upregulation of *adeR* together with increased expression of *ompA*, *carO*, and *oprD*. Although porin downregulation is classically associated with carbapenem resistance, transcriptional upregulation does not necessarily indicate increased antibiotic permeability. These patterns may reflect compensatory membrane remodeling or stress-adaptive regulatory responses rather than direct modulation of carbapenem influx. While isolate 11 additionally showed suppression of *omp33–36*, the shared transcriptional profiles of these clonally related isolates suggest that genetic background may influence the configuration of adaptive responses under antibiotic stress. In some isolates, relatively large fold-change values were observed. It should also be considered that such values may occur when baseline transcript levels are close to the detection threshold. Further investigation using higher-resolution genomic approaches may provide deeper insight into the relationship between clonal background and adaptive transcriptional patterns.

Considering all transcriptional profiles, the isolates in this study appeared to employ diverse adaptive resistance strategies in response to imipenem exposure. Because RNA was collected after prolonged sub-inhibitory exposure, some detected transcriptional patterns may reflect secondary regulatory adjustments or growth phase-associated responses rather than immediate antibiotic-triggered signaling events. Future time-course experiments with early exposure intervals are needed to distinguish primary stress responses from longer-term adaptive reprogramming. The widespread presence of *bla_OXA-23_* and *bla_OXA-24_* provides genomic potential for carbapenem hydrolysis; however, heterogeneous transcriptional regulation of outer membrane porins and efflux-associated regulators underscores the dynamic nature of the adaptive response. Frequent downregulation of permeability-associated porins such as CarO, Omp33–36, and OprD suggests a common strategy within this isolate set to limit antibiotic entry, whereas isolate-dependent *ompA* regulation may reflect membrane remodeling and stress adaptation rather than classical permeability control. In parallel, transcripts of most efflux pump structural genes (*adeA*, *adeB*, *adeC*, and *adeS*) were below the detection threshold under the tested conditions. This finding does not exclude low-level constitutive expression but suggests that strong inducible overexpression of the AdeABC system was not observed during prolonged sub-MIC imipenem exposure.

Together, these findings support the concept that carbapenem resistance in this isolate set may involve heterogeneous and potentially multifactorial transcriptional responses rather than a single dominant pathway. Under antibiotic pressure, isolates may deploy different combinations of regulatory responses and membrane permeability adjustments, potentially influenced by their genetic background. Further validation in larger and genetically diverse cohorts, coupled with integrative genomic and transcriptomic approaches, will be important to clarify the broader resistance framework.

From a clinical perspective, these findings highlight that carbapenem resistance in *A. baumannii* cannot be adequately addressed with strategies targeting a single determinant, such as carbapenemase inhibition. A more comprehensive understanding of bacterial physiology and resistance biology is needed to develop effective therapeutic strategies. New treatment strategies under investigation include novel β-lactam/β-lactamase inhibitor combinations, targeting efflux regulation and developing efflux pump inhibitors, using outer-membrane permeabilizing adjuvants to enhance intracellular antibiotic accumulation, deploying “Trojan-horse” siderophore antibiotics such as cefiderocol, exploring bacteriophage-based approaches, including phage-antibiotic synergy, and antivirulence concepts that target major outer-membrane proteins [15,31,32,33,34,35]. In parallel, antimicrobial therapy guided by molecular resistance profiling can enable more rational selection of drug combinations tailored to the specific resistance architecture of individual isolates. In this context, elucidating the regulatory and transcriptional behavior of resistance-related pathways is becoming as important as identifying the presence of resistance genes. The different adaptive responses observed among genetically diverse isolates in this study further highlight the need for integrated genomic, transcriptomic, and phenotypic analyses to capture the full complexity of carbapenem resistance in *A. baumannii*.

## 4. Materials and Methods

### 4.1. Bacterial Strains and Growth Conditions

In this study, eleven non-duplicate clinical *A. baumannii* isolates obtained from the Ankara University Faculty of Medicine, Cebeci Hospital Central Laboratory were included. The isolates were collected between January 2021 and April 2021. Identification was performed using MALDI-TOF MS (Matrix-Assisted Laser Desorption Ionization Time-of-Flight Mass Spectrometry, BioMérieux, Marcy-l’Étoile, France) (Table 3). Identification of *A. baumannii* isolates was confirmed in the laboratory using conventional methods (Figure S2). Luria–Bertani broth (LB; Merck, Darmstadt, Germany) and Luria–Bertani agar (LBA; Conda, Madrid, Spain) were used for bacterial growth. Mueller–Hinton broth (MHB; Merck, Darmstadt, Germany) and Mueller–Hinton agar (MHA; Merck, Darmstadt, Germany) were used for antibiotic susceptibility tests. *A. baumannii* ATCC 1709 (susceptible strain) and *A. baumannii* ATCC 1799 (resistant strain) were used as control strains. All test bacteria were incubated aerobically at 37 °C for 18 to 24 h. Imipenem (Sigma-Aldrich, St. Louis, MO, USA) was used in the minimum inhibitory concentration (MIC) test and the imipenem exposure step.

### 4.2. Antibiotic Susceptibility of the Clinical Isolates

Susceptibility of the clinical isolates to imipenem and meropenem was initially assessed using the disk diffusion method according to Clinical and Laboratory Standards Institute (CLSI) guidelines [36]. Standard antibiotic disks containing imipenem (10 µg) and meropenem (10 µg) (Oxoid, Basingstoke, UK) were used.

Minimum inhibitory concentrations of imipenem were subsequently determined by the broth microdilution method following CLSI recommendations [36]. Two-fold serial dilutions of imipenem, ranging from 512 to 0.25 µg/mL, were prepared in MHB. Each well was inoculated with standardized overnight bacterial cultures and incubated at 35 ± 2 °C for 18–24 h. The MIC was defined as the lowest imipenem concentration that completely inhibited visible bacterial growth. All experiments were performed in duplicate.

### 4.3. Amplification of Antibiotic Resistance Genes by Polymerase Chain Reaction (PCR)

The presence of Ade efflux pump genes (*adeA*, *adeB*, *adeC*, *adeR*, and *adeS*), outer membrane porin genes (*ompA*, *ompW*, *omp33–36*, *carO*, and *oprD*), and OXA-type carbapenemase genes (*bla_OXA-23_* and *bla_OXA-24_*) in the test bacteria was determined by polymerase chain reaction (PCR) [28,37,38,39,40,41,42,43,44]. For each target gene, a 25 μL PCR mixture was prepared, containing 12.5 μL TopTaq DNA PCR Master Mix (QIAGEN, Hilden, Germany), 1 μL of each primer (10 pmol/μL), 2.5 μL CoralLoad (QIAGEN, Germany), nuclease-free water, and template DNA. PCR amplification was performed in a Thermo Scientific Arktik Thermal Cycler (Thermo Fisher Scientific, Arktik series, Vantaa, Finland). The primer sequences are listed in Table S2, and the amplification conditions for each target gene are provided in Table S3. PCR products were separated by agarose gel electrophoresis using a 1.5% agarose gel prepared with 100 mL buffer and 5 μL SafeView™ Classic DNA stain (Applied Biological Materials Inc., Richmond, BC, Canada). Electrophoresis was performed at 85 V for 90 min, and DNA bands were visualized under UV illumination with a UV transilluminator (Bio-Rad Laboratories, Hercules, CA, USA).

### 4.4. Assessment of Sub-MIC Imipenem-Induced Transcriptional Changes in Clinical A. baumannii Isolates

Gene expression levels in *A. baumannii* isolates were quantified by reverse transcription quantitative polymerase chain reaction (RT-qPCR). After MIC determination, isolates were exposed to sub-inhibitory concentrations of imipenem (½ MIC) for 16 h under sustained antibiotic pressure in LB broth. Cultures were standardized to comparable initial optical densities before antibiotic exposure. Growth was monitored during incubation, and no substantial differences in final OD600 values were observed between imipenem-treated and untreated cultures across isolates. Therefore, no additional post-incubation normalization was performed before RNA extraction. Bacterial cells were harvested immediately before exposure and at the end of the incubation period (late exponential to early stationary phase), and cell pellets were collected for total RNA extraction and subsequent gene expression analysis.

#### 4.4.1. RNA Isolation

Total RNA was extracted using the RNeasy Mini Kit (Qiagen, Hilden, Germany) according to the manufacturer’s protocol with minor modifications. Briefly, bacterial pellets were collected by centrifugation at 5000× *g* for 5 min at 4 °C, resuspended in 700 μL RLT buffer, and vortexed vigorously. Cell disruption was enhanced by transferring the suspension to tubes containing acid-washed glass beads, followed by high-speed vortexing for 5 min. After brief centrifugation, supernatants were mixed with an equal volume of 70% ethanol and loaded onto RNeasy spin columns. Sequential washing steps were performed using RW1 and RPE buffers, and RNA was eluted in 50 μL of nuclease-free water. RNA concentration and purity were measured using a NanoDrop spectrophotometer (Thermo Fisher Scientific, Waltham, MA, USA) and RNA integrity was assessed by agarose gel electrophoresis.

#### 4.4.2. cDNA Synthesis

Complementary DNA (cDNA) was synthesized from the isolated RNA using the iScript cDNA Synthesis Kit (Bio-Rad Laboratories, Hercules, CA, USA), following the manufacturer’s instructions. Reaction components and thermal conditions are provided in Table 4.

#### 4.4.3. Reverse Transcription Quantitative Polymerase Chain Reaction (RT-qPCR)

Quantitative real-time PCR analyses were performed using the iTaq Universal SYBR Green Supermix (Bio-Rad Laboratories, USA) on a CFX96 Touch Real-Time PCR Detection System (Bio-Rad Laboratories, USA). Each reaction was prepared at a final volume of 10 μL. Primer sequences are listed in Table S2, and thermal cycling conditions are provided in Table 5. Total RNA samples were treated with DNase before cDNA synthesis to minimize genomic DNA contamination. Negative reverse transcription controls (no-RT controls) were included to confirm the absence of genomic DNA amplification.

Samples collected before imipenem exposure were designated as the control condition, while samples collected after sub-MIC exposure constituted the treatment condition. Gene expression data were normalized to the 16S rRNA housekeeping gene. Relative expression was calculated using the 2^(−ΔΔCt)^ method, where ΔCt = Ct(target gene) − Ct(reference gene) and ΔΔCt = ΔCt(treatment) − ΔCt(control). A Ct value greater than 35 was considered below the detection threshold. Targets that did not produce a specific amplification curve within 35 cycles were classified as undetectable under the tested conditions. For presentation, relative expression values were log_2_-transformed to improve the stability and interpretability of fold-change estimates. Results are reported descriptively as log_2_ fold-change values derived from technical duplicates.

### 4.5. Determining Clonal Relatedness by Arbitrarily Primed PCR (AP-PCR)

The clonal relationships of the isolates were determined using arbitrarily primed polymerase chain reaction (AP-PCR). The M13 primer (F: 5′-GTAAAACGACGGCCAGTGAA-3′, R: 5′-AACAGCTATGACCATGA-3′) was used [45]. A 25 μL PCR mixture was prepared with the TopTaq DNA PCR Master Mix Kit (Qiagen, Germany). The PCR was performed in a thermocycler as follows: pre-denaturation at 94 °C, eight cycles of denaturation at 94 °C for 30 s, annealing at 37 °C for 1 min, and extension at 72 °C for 2 min; 35 cycles of denaturation at 94 °C for 30 s, annealing at 60 °C for 1 min, and extension at 72 °C for 2 min; and a final extension at 72 °C for 5 min. PCR products were electrophoresed in a 2% agarose gel with 1 × TAE buffer, initially at 100 V for 1 h, then at 50 V for 8 h. Band profiles were analyzed using GelCompar II software (version 6.6; Applied Maths, Sint-Martens-Latem, Belgium). The Dice correlation coefficient was used to calculate similarity in band analysis, and the Unweighted Pair Group Method with Arithmetic Mean (UPGMA) was used for cluster analysis (optimization 1.0, tolerance 1.0). Isolates with more than 80% similarity between bands were considered the same clone.

## 5. Conclusions

This preliminary study provides initial insight into the heterogeneous transcriptional responses of clinical *A. baumannii* isolates exposed to imipenem. Within this isolate set, carbapenem resistance appears to involve isolate-specific combinations of regulatory and permeability-associated responses rather than reliance on a single dominant mechanism. The observed variability in porin and efflux regulator expression patterns underscores the complexity of adaptive responses under antibiotic stress. Further validation in larger and genetically diverse cohorts, together with integrative genomic and transcriptomic approaches, will be necessary to define the broader resistance architecture and its clinical implications. Such integrative approaches will be essential for unraveling the pathogen’s resistance mechanisms and developing next-generation treatment strategies.

## Figures and Tables

**Table 1:** Imipenem MIC values of clinical isolates and reference strains.

Isolate Number	Clinical Samples	MIC Values (μg/mL)
1	Blood	16
2	Sputum	32
3	Sputum	32
4	Sputum	32
5	Tissue	32
6	Tracheal aspirate	8
7	Urine	16
8	Sputum	16
9	Tissue	64
10	Urine	64
11	Urine	32
*A. baumannii* ATCC 1709		<0.25
*A. baumannii* ATCC 1799		16

MIC values were determined by broth microdilution according to CLSI guidelines. Isolates with MIC ≥ 4 µg/mL were classified as imipenem-resistant. *A. baumannii* ATCC 1709 and *A. baumannii* ATCC 1799 were included as quality control strains.

**Table 2:** Relative expression levels of resistance-associated genes in *A. baumannii* isolates before and after exposure to imipenem.

Isolate	Condition	*adeR*	*ompA*	*carO*	*oprD*	*omp33–36*
1	Pre *	–	1.015	1.018	1.156	–
	Post	–	0.529	0.874	15.67	–
2	Pre *	1.037	1.025	1.020	–	–
	Post	449.368	1.035	0.491	–	–
3	Pre *	1.000	1.018	1.024	1.051	–
	Post	0.036	0.038	0.046	0.032	–
4	Pre *	1.001	1.001	1.018	–	–
	Post	0.016	0.010	0.031	–	–
5	Pre *	1.142	1.000	1.032	1.148	–
	Post	0.041	0.021	0.009	0.003	–
6	Pre *	1.052	1.007	1.038	1.002	–
	Post	0.0001	0.002	0.001	0.00067	–
7	Pre *	1.001	1.025	1.012	1.030	–
	Post	1.882	0.986	0.764	0.606	–
8	Pre *	–	1.049	1.042	1.063	–
	Post	–	0.450	0.285	0.403	–
9	Pre *	1.002	1.006	1.019	1.113	–
	Post	0.098	0.221	0.157	0.092	–
10	Pre *	1.170	1.006	1.002	1.003	–
	Post	939.649	917.546	992.313	26.575	–
11	Pre *	1.001	1.024	1.001	1.018	1.007
	Post	24.450	18.139	23.516	49.183	0.176

Relative gene expression levels were calculated using the 2^−ΔΔCt^ method and normalized to the 16S rRNA housekeeping gene. Pre-exposure samples (*) served as calibrators. A dash (–) indicates undetectable transcript levels under the tested conditions. Notably, *adeA*, *adeB*, *adeC*, *adeS*, *ompW*, *bla_OXA-23_*, and *bla_OXA-24_* transcripts were not detected in any isolate, despite their confirmed genetic presence.

**Table 3:** *A. baumannii* isolates and their clinical sources.

Isolate Number	Clinical Source
1	Blood
2	Sputum
3	Sputum
4	Sputum
5	Tissue
6	Tracheal aspirate
7	Urine
8	Sputum
9	Tissue
10	Urine
11	Urine

**Table 4:** Reaction components prepared for quantitative reverse transcription PCR (qRT-PCR).

Reagent	Volume (µL)
iTaq™ Universal SYBR^®^ Green Supermix (2×)	5.0
Forward primer	0.5
Reverse primer	0.5
Template cDNA	Variable
Nuclease-free water	Variable
Total volume	10

**Table 5:** Quantitative real-time PCR (qRT-PCR) thermal cycling.

Step	Temperature (°C)	Time	Cycles	Description
Initial activation/denaturation	95	30 s	1	Polymerase activation and DNA denaturation
Denaturation	95	15 s	40	DNA strand separation
Annealing *	47–59	30 s	40	Primer binding
Extension	72	30 s	40	DNA synthesis

* Annealing temperature varied depending on the primer set used.

## Data Availability

The data presented in this study are available within the article and its Supplementary Materials.

## Supplementary Materials

The following supporting information can be downloaded at https://www.mdpi.com/article/10.3390/antibiotics15030299/s1, Figure S1: Agarose gel electrophoresis profiles showing PCR amplification of resistance- and permeability-associated genes in the isolates; Figure S2: Identification of *A. baumannii* isolates with conventional methods; Table S1: Antibiotic susceptibility of the *A. baumannii* isolates; Table S2: Primers used for amplification of antibiotic resistance genes; Table S3: PCR amplification conditions for antibiotic resistance genes.
