# Supplementary material for: Imipenem-Induced Transcriptional Responses of Porin, Efflux Pumps, and Carbapenemase Genes in Clinical Carbapenem-Resistant Acinetobacter baumannii
Source: Antibiotics (Basel). 2026 Mar 15;15(3):299. doi: 10.3390/antibiotics15030299 (PMC13023831; doi:10.3390/antibiotics15030299)
Supplement: Supplementary file 1 [file antibiotics-15-00299-s001.zip › antibiotics-4158978-supplementary.pdf]

Table S1. Antibiotic susceptibility of the *A. baumannii* isolates

Isolate Number	Imipenem	Meropenem
**1**	— (R)	— (R)
**2**	— (R)	— (R)
**3**	— (R)	— (R)
**4**	— (R)	— (R)
**5**	— (R)	— (R)
**6**	— (R)	— (R)
**7**	— (R)	— (R)
**8**	— (R)	— (R)
**9**	— (R)	— (R)
**10**	— (R)	— (R)
**11**	— (R)	— (R)

—; No zone of inhibition, inhibition zones were measured in "mm".,S; susceptibility, I; intermediate, R; resistant. For imipenem, ≥ 22 (S) - 19–21 (I) - ≤ 18 (R); for meropenem, ≥ 18 (S) - 15–17 (I) - ≤ 14 (R) (CLSI).

Table S2. Primers used for amplification of antibiotic resistance genes

Gene	Primer	Primer Sequence (5′–3′)	Reference
adeA	F	ATCTTCCTGCACGTGTACAT	[37]
	R	GGCGTTCATACTACTAACG	[37]
adeB	F	GTATGAATTGATGCTGC	[37]
	R	CACTCGTAGCCAATACC	[37]
adeC	F	AGCCTGCAATTACATCTAT	[37]
	R	TGGCATCTACTATCAATAC	[37]
adeS	F	TGCCGCCAAATTCTTTATTC	[38]
	R	TTAGTCACGGCGACCTCTCT	[38]
adeR	F	CGCTCTAGTGCATCGCTATC	[38]
	R	GCATTACGCATAGGTGCAGA	[38]
ompA	F	TCTTGGTGGTCACTTGAAGC	[39]
	R	ACTCTTGTGGTTTGTGGAGCA	[39]
ompW	F	TATGGATCCGGTAATTGGCAAGTAAAATTTGGG	[28]
	R	TATAAGCTTTTAGAATTTATAGCTATAGCC	[28]
carO	F	AGCTTTACTTGCTGCTGGTG	[40]
	R	CGAGCGCCTACTGAATTA	[40]
omp33–36	F	CAAGTGTTGCTAACCAATTCGCT	[41]
	R	GTTTTCTTGACCGATGCACC	[41]
oprD	F	CCAGCTCAGTTGCTCAATCA	[42]
	R	CATTTGGTTTCCAGCGTTTT	[42]
bla$_{\text{OXA-23}}$	F	CCTCAGGTGTGCTGGTTATTCA	[43]
	R	CTCCAATCCGATCAGGGCAT	[43]
bla$_{\text{OXA-24}}$	F	GCAGAAAGAAGTAAAGCGGGTTA	[43]
	R	AGGTAATCGGTTATGTGCAAGGT	[43]
16S rDNA	F	TCAGCTCGTGTCGTGAGATG	[44]
	R	CGTAAGGGCCATGATG	[44]

F: Forward primer; R: Reverse primer
Primer sequences are written in the 5–3′ direction.

Table S3. PCR amplification conditions for antibiotic resistance genes

Target genes	Cycle step	Time	Temperature (°C)
adeA, adeB, adeC, adeS, adeR, ompA, ompW, carO, omp33–36, oprD, $bla_{\text{OXA-23}}$, $bla_{\text{OXA-24}}$, *16S rDNA*	Initial denaturation	3 min	95
	Denaturation (35 cycles)	30 s	95
	Annealing*	30 s	47-59
	Extension	1 min	72
	Final extension	10 min	72

**Annealing temperature varied depending on the primer set used.*

(a)

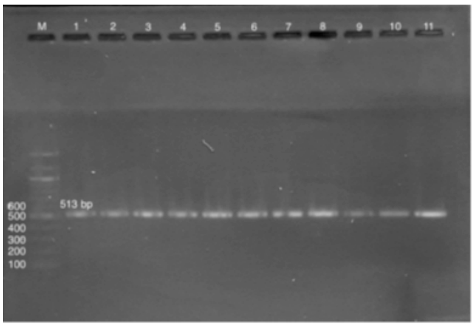

(b)

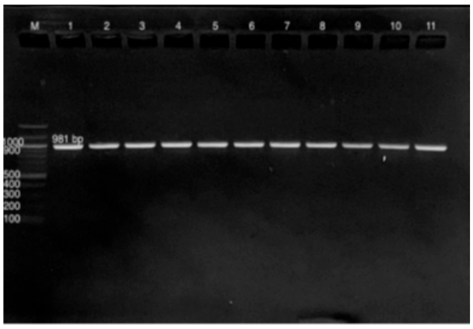

(c)

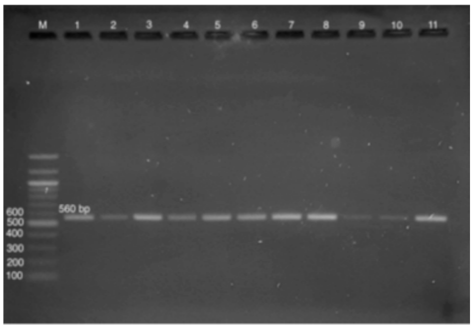

(d)

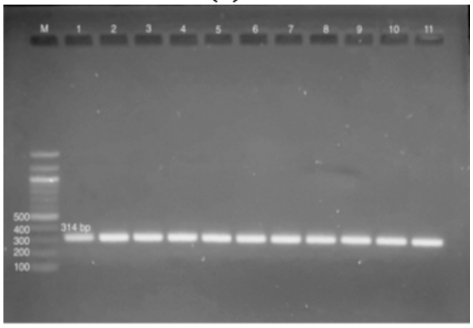

Figure S1. Agarose gel electrophoresis profiles showing PCR amplification of resistance- and permeability-associated genes in the isolates. Panels show amplification of **(a)** *adeA* (513 bp), **(b)** *adeB* (981 bp), **(c)** *adeC* (560 bp), **(d)** *adeR* (314 bp). Lane M indicates the 100 bp DNA ladder, and lanes 1-11 represent the tested isolates

(e)

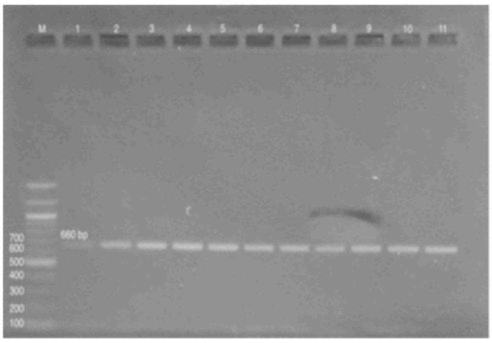

(f)

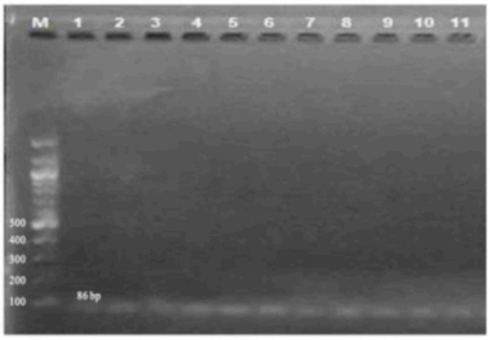

(g)

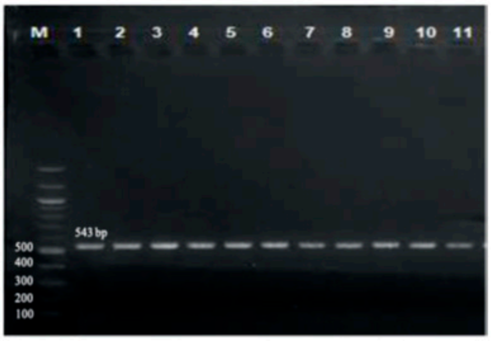

(h)

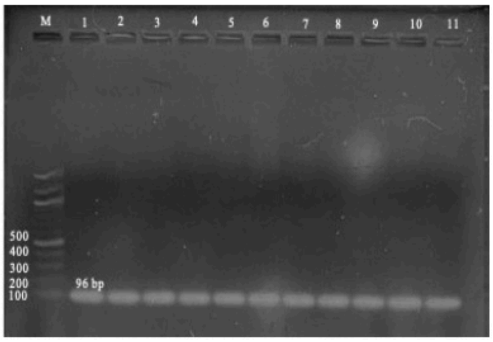

Figure S1. Continued (e) *adeS* (660 bp), **(f)** *ompA* (86 bp), **(g)** *ompW* (543 bp), **(h)** *carO* (96 bp). Lane M indicates the 100 bp DNA ladder, and lanes 1-11 represent the tested isolates

(i)

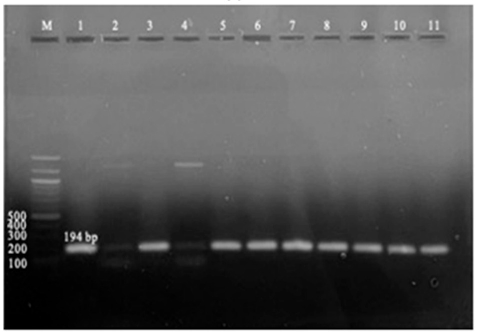

(j)

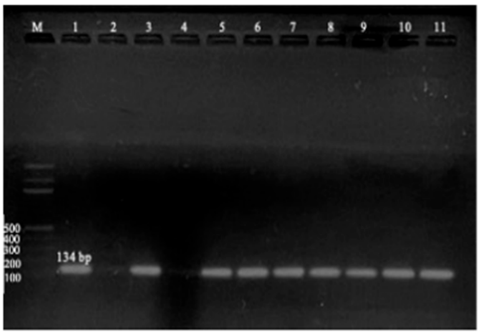

(k)

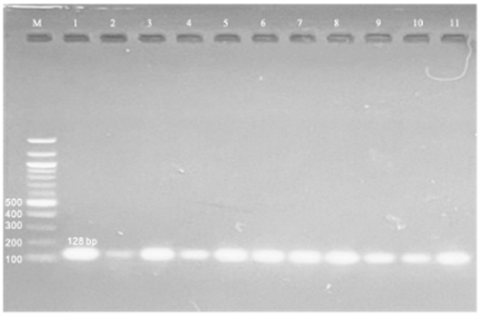

(l)

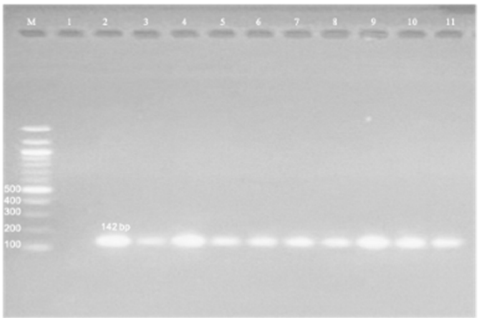

Figure S1. Continued (i) *omp33-36* (194 bp), **(j)** *oprD* (134 bp), **(k)** *bla_{OXA-23}* (128 bp), and **(l)** *bla_{OXA-24}* (142 bp). Lane M indicates the 100 bp DNA ladder, and lanes 1-11 represent the tested isolates. The *bla_{OXA}-24* gene was not detected in isolate 1

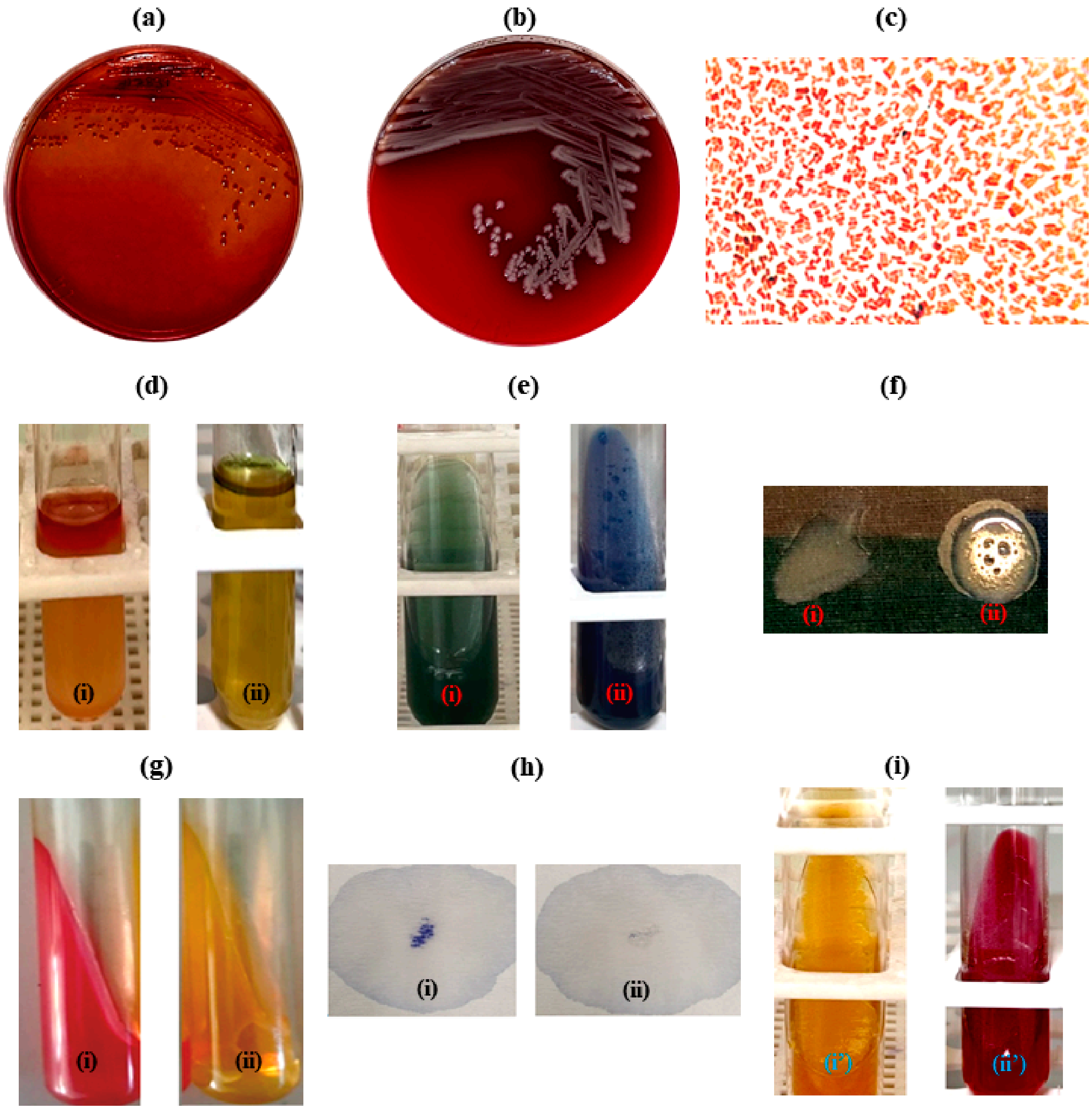

Figure S2. Identification of *A. baumannii* isolates with conventional methods. **(a)** Lactose-negative *A. baumannii* colonies on MacConkey agar and **(b)** non-hemolytic, opaque *A. baumannii* colonies on blood agar. **(c)** Gram-negative coccobacillary morphology observed by Gram staining. **(d)** Indole test: positive reaction in *E. coli* ATCC 25922 **(i)** and negative reaction in *A. baumannii* isolates **(ii)**. **(e)** Citrate test: negative reaction in *E. coli* ATCC 25922 **(i)** and positive reaction in *A. baumannii* isolates **(ii)**. **(f)** Catalase-positive reaction in *A. baumannii* isolates **(ii)**. **(g)** Urease test: positive reaction in *K. pneumoniae* ATCC 13883 **(i)** and negative reaction in *A. baumannii* isolates **(ii)**. (h) Oxidase test: positive reaction in *P. aeruginosa* ATCC 27853 **(i)** and negative reaction in *A. baumannii* isolates **(ii)**. **(i)** Triple Sugar Iron (TSI) agar test: positive sugar fermentation in *E. coli* ATCC 25922 **(i′)** and negative reaction in *A. baumannii* isolates **(ii′)**
